# Concurrent loss of MLH1, PMS2 and MSH6 immunoexpression in digestive system cancers indicating a widespread dysregulation in DNA repair processes

**DOI:** 10.3389/fonc.2022.1019798

**Published:** 2022-10-26

**Authors:** Nic Gabriel Reitsam, Bruno Märkl, Sebastian Dintner, Johanna Waidhauser, Dmytro Vlasenko, Bianca Grosser

**Affiliations:** ^1^ General Pathology and Molecular Diagnostics, Medical Faculty, University of Augsburg, Augsburg, Germany; ^2^ Department of Hematology and Oncology, University Medical Center Augsburg / University Hospital of Augsburg, Augsburg, Germany; ^3^ General, Visceral and Transplantation Surgery, University Hospital of Augsburg, Augsburg, Germany

**Keywords:** digestive system cancer, gastrointestinal cancer, DNA Repair, MSI, mismatch-repair proteins, homologous recombination repair

## Abstract

Immunohistochemical analysis of mismatch repair (MMR) protein expression is widely used to identify tumors with a deficient MMR (dMMR). MMR proteins (MLH1/PMS2 and MSH2/MSH6) work as functional heterodimers, which usually leads to the loss of expression in only one functional MMR heterodimer. Recently, there have been studies showing the simultaneous loss of immunoexpression in proteins of both heterodimers. Yet, this phenomenon has been rarely investigated. In this study, we retrospectively considered cases of different digestive system cancers (gastric cancer, ampullary cancer, small bowel cancer, colorectal cancer), which were immunohistochemically tested for dMMR within a 4-year period at our university hospital (n=352). Of the 103 cases showing dMMR, 5 cases (1.4% of all, 5.1% of dMMR cases) showed a concurrent loss of MLH1, PMS2 and MSH6 immunoexpression, whereas in the other 98 dMMR cases only one MMR heterodimer was affected. MLH1^-^/PMS2^-^/MSH6^-^ cancer cases almost arose throughout the entire digestive tract: from the gastric antrum to the left colic flexur. To provide a comprehensive molecular characterization of this MLH1^-^/PMS2^-^/MSH6^-^ immunophenotype, tumors were analyzed for microsatellite instability, *MLH1* promotor hypermethylation and *BRAF* exon 15 status. Furthermore, we performed next-generation sequencing focusing on genes related to DNA repair. Here, we could detect pathogenic germline variants as well as multiple sporadic mutations in different genes involved in MMR and homologous recombination repair (HRR) respectively. The affected MMR/HRR-related genes were: *ATM, BARD1, BRCA1, CDK12, CHEK1, CHEK2, FANCA, MLH1, MSH6, PALB2, TP53*. Considering the biologic function of HRR/MMR proteins as potential drug targets and the low frequency of most of these mutations in digestive system cancers in general, their common occurrence in our MLH1^-^/PMS2^-^/MSH6^-^ cases seems to be even more noteworthy, highlighting the need for recognition, awareness and further investigation of this unusual IHC staining pattern.

## Introduction

DNA mismatch repair deficiency (dMMR) is routinely studied by immunohistochemical staining *via* analyzing the expression of MMR proteins (MLH1/PMS2 and MSH2/MSH6). As MLH1/PMS2 and MSH2/MSH6 work together as functional heterodimers (1) and as PMS2 and MSH6 are unstable in absence of MLH1 and MSH2 respectively, there is usually only one DNA MMR subsystem affected in malignancies with dMMR. However, there exist also rare unusual cases with defects in both MMR subsystems, which have been described in recent studies (2–6) and coincide with our clinical practice. Furthermore, there are also digestive system cancer cases that show a heterogenous loss of MMR protein expression, which is also a rarely studied phenomenon (7–9).

With MLH1 promoter hypermethylation being the most common reason for dMMR (10), the most widespread immunohistochemical pattern is the loss of MLH1 and PMS2 expression (11). Simultaneous loss of MLH1 and PMS2 expression is also the most common pattern in Lynch syndrome based on MLH1 germline mutations, followed by a MSH2/MSH6 loss due to MSH2 germline mutations. In much lower frequency, isolated losses of PMS2 (12) or MSH6 (8, 13) occurred in patients with LS due to the corresponding germline mutations. As cells that are unable to correct DNA replication mistakes accumulate errors, dMMR induces microsatellite instability (MSI). Typically, dMMR and MSI-high (MSI-H) are used nearly interchangeably, as there is a high level of consistency (14). Plenty is known about the frequency, aetiology, prognostic (15, 16) and predictive (17, 18) implications of dMMR/MSI-H status. However, there are only few studies focusing on the special subgroup of malignancies with an unusual immunophenotype, namely the loss of expression in both MMR functional heterodimers (MLH1/PMS2 and MSH2/MSH6) (2-6). In general, MSI is a frequent phenomenon in different digestive tract cancer entities like colorectal carcinoma (CRC) or gastric cancer (GC), which both contribute extensively to the burden of disease globally (19). Thus, we included four different digestive tract cancers: small bowel carcinoma, CRC, GC and ampullary cancer. These cancer entities have very different prevalences but all display a significant proportion of MSI. For small bowel carcinoma widely differing MSI rates exist in the literature: from approximately 23% (20) up until 45.5% (21). GCs display a MSI frequency of 9-19% and CRCs of 6-19% (22). Finally, ampullary cancers possess a MSI frequency of about 6% (23) to 10.4% (24). Whereas MMR acts during DNA replication to correct for polymerase errors, MMR is also closely linked to homologous recombination repair [HRR (25)], a mechanism, that constantly repairs double-strand breaks in DNA. Considering this overlap of MMR and HRR and with the advent of drugs targeting DNA repair in clinical practice for other cancer entities, especially PARP-inhibitors for *BRCA*-mutated carcinomas (26, 27), we proposed that our subgroup with a dysregulated MMR immunophenotype may be also a suitable cohort to search for additional defects in DNA repair processes. As novel therapies targeting other HRR pathway components than *BRCA1/*2 like *ATM*, *ATR* or *CHEK1/2* are now under clinical investigation (28, 29), working out subgroups likely to benefit from such drugs will be crucial.

We aim to shed light on features of this peculiar phenotype with an involvement of both MMR subsystems. Therefore, the aim of this study was a comprehensive molecular characterization of digestive system cancer cases with concurrent, immunohistochemically detectable deficiencies in both DNA MMR subsystems. Besides analyzing the frequency, providing clinical characteristics and performing conventional histology, immunohistochemistry, PCR-based MSI testing, testing for *MLH1* promotor hypermethylation and *BRAF* exon 15 mutation, we additionally applied next-generation sequencing (NGS) focusing on genes related to DNA repair to further understand the molecular basis of dysregulated MLH1/PMS2 and MSH2/MSH6 expression among different digestive system cancer entities.

## Material and methods

### Ethical approval and patient cohort

The study was approved by the ethical committee of Ludwig Maximilian University (LMU) of Munich (reference: project number 22-0381) and was performed in accordance with the Declaration of Helsinki. Prior to enrolling cases with an involvement of both MMR subsystems into the study and performing further molecular testing, the affected patients gave written informed consent.

The entire cohort to identify cases of interest consisted of 352 cases, that underwent immunohistochemical testing for dMMR at the University Hospital Augsburg between January 2018 and February 2022 (4-year period) with the two-stain method, initially only testing for PMS2 and MSH6 expression (30, 31). In case that both markers were negative initially (PMS2^-^, MSH6^-^), the two missing MMR markers (MLH1, MSH2) were always stained additionally. The cohort was assembled by a retrospective database search of our institution’s internal laboratory information system. As stated, we included different digestive system cancer entities each with a considerable MSI frequency: 300 CRCs, 41 GCs, 9 ampullary cancers and 2 small bowel carcinomas. As the simultaneous deficiency in both MMR subsystems is a rare event – presumably about 1-5% (3, 5, 6), we tried to study a broad variety of cases in order not to miss any cases. Hence, we included a) primary cancers as well as metastases, b) biopsies as well as resected specimens, c) patients with and without neoadjuvant chemotherapy, d) different tumor stages (T1 to T4), and e) different histologic subtypes e.g. in CRC mucinous, medullary or signet-cell, in GC intestinal or diffuse and in ampullary cancer pancreatobiliary as well as intestinal type. Patients had a median age of 70 years (25% quantile: 61; 75% quantile: 79) with a balanced male/female-ratio of 175/177. After informed consent, we completed molecular testing (if missing: MSI-PCR, *MLH1* promotor methylation and/or *BRAF* status) on the formalin-fixed paraffin-embedded (FFPE) material from these peculiar cases with a dysregulated expression in both MMR subsystems, and additionally performed NGS with a focus on HRR genes.

### Immunohistochemistry

Immunohistochemical (IHC) staining was performed on 2 µm whole slides sections using primary antibodies for MLH1 (Clone: M1, Roche [Basel, Switzerland], RTU), PMS2 (Clone: EP51, Agilent Technologies [Santa Clara, CA, USA], RTU), MSH2 (Clone: G219-1129, Roche [Basel, Switzerland], RTU) and MSH6 (Clone: EP49, Leica Biosystems [Newcastle, UK], RTU) in a fully automated manner on a Ventana BenchMark ULTRA platform with an iVIEW DAB detection system (Roche, Mannheim, Germany). Nuclear staining throughout the whole tumor that is predominantly stronger in intensity than that of the internal control was considered as proficient MMR (pMMR). Any deviation from this staining pattern constitutes an abnormal pattern; especially the loss of nuclear expression of MSH6 or PMS2 is typical for dMMR cases. Partial or heterogeneous loss of expression was defined according to the criteria published by Joost et al. as tumors showing either zonal loss or so-called intraglandular heterogeneity (9). A strong homogeneous nuclear staining of non-neoplastic cells served as an internal control. Adequate controls were used for quality control of staining. All IHC slides as well as conventional H&E stains were digitized using a 3D Histech Pannoramic Scan II.

### MLH1 promotor methylation status

DNA was extracted from paraffin-embedded tissue by using the Maxwell^®^ FFPE Plus DNA Kit (Promega, AS1135 [Madison, Wisconsin, USA]) following the manufacturer’s protocol. DNA methylation patterns in the CpG islands of *hMLH1* gene was determined by chemical treatment with sodium bisulfite (Zymo EZ DNA Methylation Kit #D5001) and subsequent methylation-specific polymerase chain reaction (MSP, MS-PCR) as described (32). Primer sequences for *hMLH1* for unmethylated reaction were 5’-AGAGTGGATAGTGATTTTTAATG-3’ (forward) and 5’-CCTCATACTCACATTCTTCCT-3’ (reverse), and for the methylated reaction were 5’-AGCGGATAGCGATTTTTAACG-3’ (forward) and 5’-AAACGTCTAAATACTCAACGAAA-3’ (reverse). All PCRs were performed with positive controls for both unmethylated and methylated alleles and no template control. The amplified fragments were separated on a 1.5% agarose gel. Here, PCR yields a 202 bp amplicon in the presence of a hypomethylated *MLH1* promoter. In the presence of a hypermethylated *MLH1* promoter, a 248 bp product is amplified.

### Microsatellite analysis

For MSI molecular test was performed on normal and tumor DNA extracted from paraffin-embedded tissue by using the Maxwell^®^ FFPE Plus DNA Kit (Promega, AS1135) following the manufacturer’s protocol. Corresponding normal and tumor DNA were investigated with a set of 5 microsatellite markers (BAT25, BAT26, D5S346 [*APC* locus], D17S250 [*p53* locus] and D2S123) by multiplex amplification. These were subsequently separated by capillary electrophoresis using a SequStudio Genetic Analyzer (Applied Biosystems, Waltham, Massachusetts, USA), and analyzed by GeneMapper software v4.1 (Applied Biosystems). The performed examinations and analysis of the data were performed according to the guidelines of the study protocol of the collaborative project “Familial Colorectal Cancer” of the German Cancer Aid and correspond to point 5 of the guidelines for HNPCC (Hereditary Non Polyposis Colorectal Carcinoma) clarification (www.krebshilfe.de). MSI was defined as any marker with the highest peak shifted more than two base pairs when compared to the same marker in the normal sample. MSI-H (MSI-high) is present if at least 40% of the microsatellites of the biomarkers (2 or more) of the NCI panel (BAT25, BAT26, D2S123, D5S536, D17S250) show microsatellite instability (33).

### Sanger sequencing

Sanger sequencing was performed on tumor samples using 50 ng of genomic DNA with 0.83 µM of each primer, 0.6 U Ampli-Taq GOLD (Thermo Fisher Scientific, Waltham, Massachusetts, USA), 10 × Buffer II (Thermo Fisher Scientific) an 0.2 mM each dNTP. Cycling conditions were denaturation an 95°C for 10 min, followed by 40 cycles of 30 s 94°C, 30 s of 60°C and 30 s of 72°C, with final elongation at 72°C for 10 min.

Sanger sequencing was performed using the BigDye Terminator v1.1 Cycle Sequencing Kit (Thermo Fisher Scientific) and analyzed using Sequencing Analysis Software (Applied Biosystems). Primers used to amplify *BRAF* exon 15 were 5’-CATAATGCTTGCTCTGATAGGAAAATG-3’ (forward) and 5’-CATCCACAAAATGGATCCAGACA-3’ (reverse).

### NGS HRR Panel

NGS was performed on normal and tumor tissue. For DNA extractions from FFPE samples, tumor areas were marked on hematoxylin–eosin (H&E) stained slides by a pathologist. The corresponding area was macrodissected from two to three 10 µm sections with at least 20% tumor cellularity. For the library preparation of this study the multiplex PCR based AmpliSeq for Illumina Custom Panel (HRR IPMD; Illumina [San Diego, CA, USA]) was used (Custom designed panel, this panel is available for research use only). The panel consists of 1294 primer pairs for the detection of hot-spot regions and full-genes in 25 HRR-related genes with a targeted size of 101654 bp. Amplicon library preparation was performed using approximately 10 to 100 ng of DNA, as recommended by the manufacturer. In brief, the DNA was mixed with a primer pool 1 and 2 containing all primers for generating the amplicons and with the AmpliSeq HiFi master mix. The mastermix was transferred to a PCR cycler. PCR cycling conditions were initial denaturation at 99°C for 2 minutes, followed by 21 cycles of 99°C for 15 seconds and 60°C for 4 minutes. After the end of the PCR reaction, primer end sequences were partially digested using FuPa reagent according to the manufacturer’s instructions; this step was followed by the ligation of barcoded sequencing adapters (AmpliSeq CD Indexes Set A, for Illumina Technologies). The final library was purified using AMPure XP magnetic beads (BeckmanCoulter, Krefeld, Germany) and was quantified using QuantiFluor^®^ ONE dsDNA System (Promega) The individual libraries were diluted to a final concentration of 11 pmol/L, and libraries were pooled and processed for sequencing by synthesis using a MiSeq reagent kit V2 (300 cycles) on a MiSeq System (Illumina).

*Data analysis*: Secondary analysis was performed using the application Generate FASTQ (Version 2.0.01.17; RUO) and IAA26013167 manifest (Version 1; RUO) on the Local Run Manager (Version 1.0.0.7; Illumina). The reads were aligned to the human reference sequence build hg19. Detection of SNVs and indel polymorphisms, relative to the human reference sequence, was performed using the BaseSpace Variant Interpreter (Illumina). Filtering was performed for non-synonymous and non-polymorphic alterations. The detection limit of variant allele frequency is 1%. In addition, the data were analyzed using the BaseSpace Knowledge Network and the variants were documented accordingly. Interpretation of the alterations was performed using different databases according to ACMG guidelines (34), such as cbioportal.org, ClinVar, Varsome, and CiVIC. Classification of BRCA1/2 mutations is based on the six public databases ARUP (Association of regional and University Pathologists Inc.), BRCA Exchange, 5.2AC (Instituté Recherche Association Cancer), LOVD (Leiden Open Variation Database), NIH (National Institutes of Health), and UMD (Universal Mutation Database).

*Analyzed genes:* The gene list of the AmpliSeq Illumina HRR IPMD contains the following genes: *ATM, BARD1, BRCA1, BRCA2, BRIP1, CDK12, CHEK1, CHEK2, FANCA, FANCL, NBN, PALB2, RAD51B, RAD51C, RAD51D, RAD54L, TP53, FANCC, FANCG, FANCD2, MSH2, MSH6, MLH1, PMS2*, and *EPCAM.*

## Results

### Frequency of concurrent loss of MLH1/PMS2 and MSH6 expression among different digestive system cancer entities

**Figure 1:**
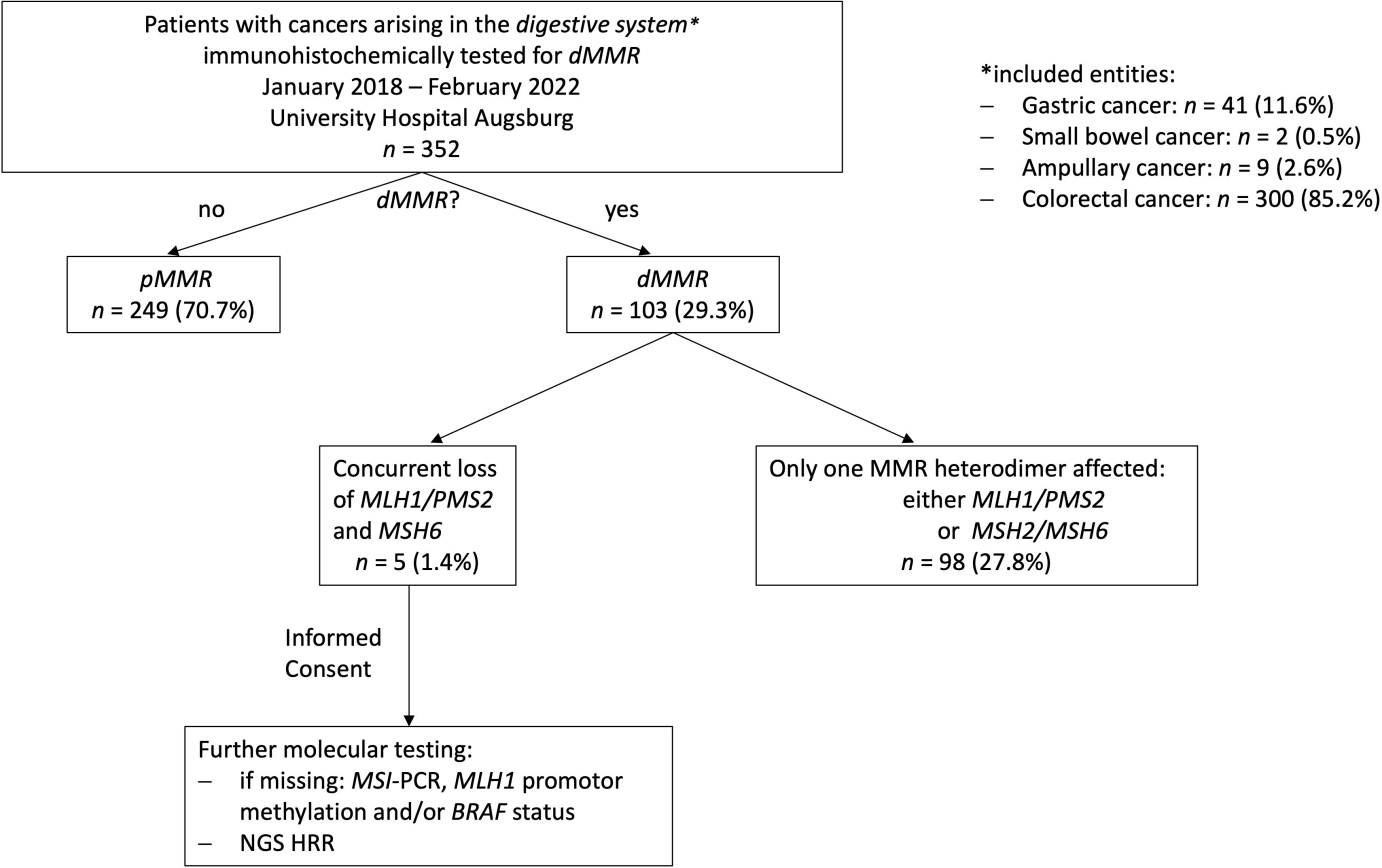
Study flow chart. 352 digestive system cancer samples underwent immunohistochemical testing for MMR deficiencies over a 4-year period at our hospital (University Hospital Augsburg, Germany). Only, five MLH1^-^/PMS2^-^/MSH6^-^ could be identified. Consecutively, additional extensive molecular testing including a NGS panel focusing on genes involved in MMR/HRR was applied on these cases.

A total of 352 cases of digestive system cancers from January 2018 to February 2022 from Augsburg University Hospital, for which immunohistochemical testing for MMR proteins was routinely performed in clinical practice, were retrospectively analyzed. Whereas 249 (70.7%) cases showed a proficient DNA MMR (pMMR), 103 cases (29.3%) displayed deficiencies in DNA MMR. In the vast majority of dMMR cases (n=98), only one MMR heterodimer, either MLH1/PMS2 or MSH2/MSH6, was affected. However, overall five cases (1.4% of all cases, 5.1% of cases with dMMR) presented with a concurrent loss of MLH1/PMS2 and MSH6. Interestingly, we could only observe cases with a concurrent loss of MLH1/PMS2 and MSH6 expression and no other immunophenotype with an involvement of both functional MMR heterodimers. The whole study sequence is shown in the patient flow chart in **Figure 1**.

### Clinicopathological findings

**Figure 2:**
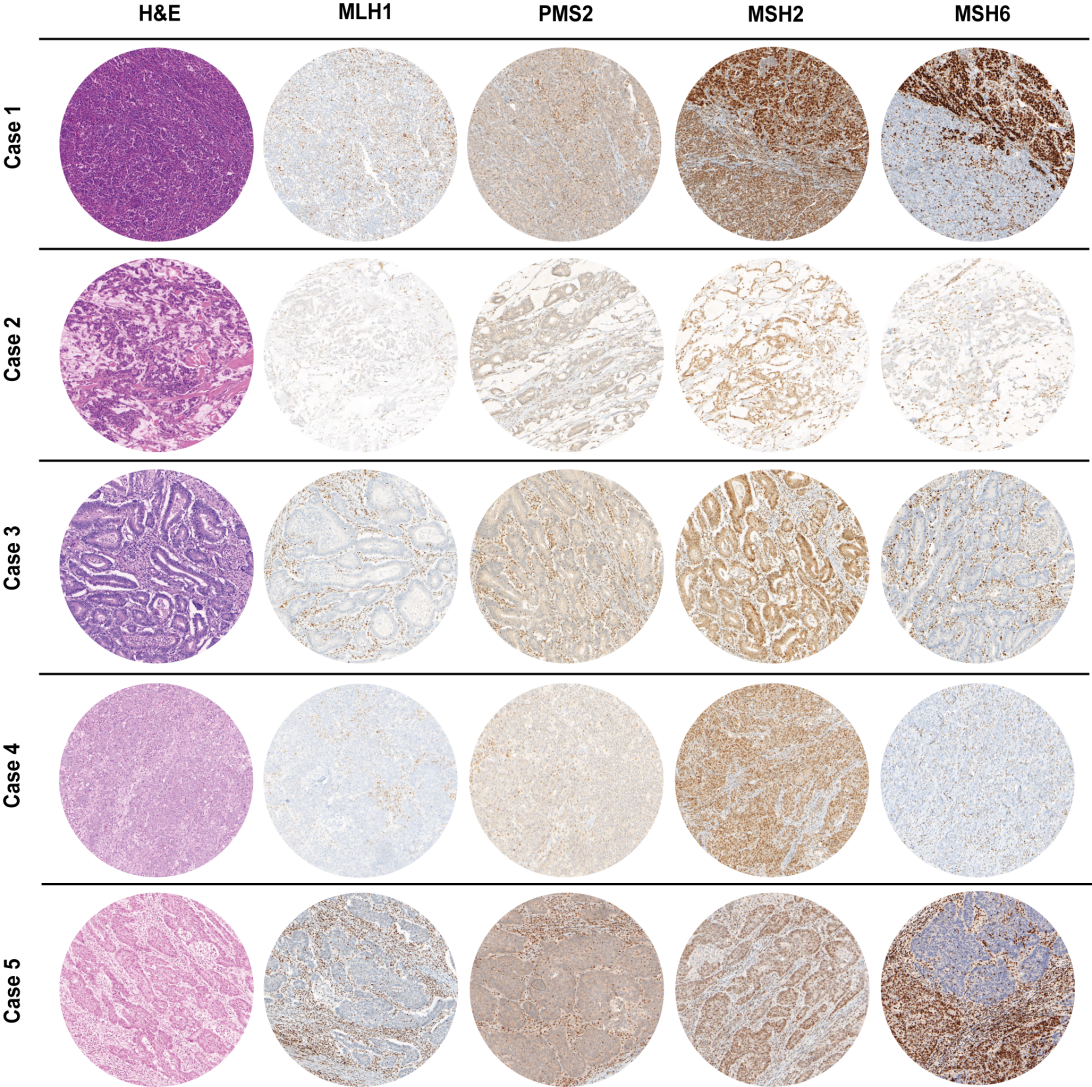
H&E as well as MMR IHC of MLH1^-^/PMS2^-^/MSH6^-^ cancer cases arising in the digestive tract (100x magnification). Whereas MSH2 staining was preserved, MLH1 & PMS2 were completely absent in all cases. Cases 1&5 presented with a heterogeneous staining pattern for MSH6 immunoexpression with only a partial loss. In all IHC slides, nuclear staining of stromal cells as well as infiltrating lymphocytes served as internal positive control.

The clinicopathological features of the five MLH1^-^/PMS2^-^/MSH6^-^ cases are summarized in **Table 1**. Most patients (4 out 5, 80%) were female, with ages at diagnosis ranging from 63 to 82 years. The sites of cancer origin were distributed throughout the digestive tract (gastric antrum, ampulla vateri, caecum, colon transversum, left colic flexur). We could not identify any rectal cancer with a concurrent MLH1/PMS2 and MSH6 loss. Only one included case (case 5) received neoadjuvant therapy prior to resection. Regarding TNM classification, the five cases were heterogenous with T-stages ranging from 2 to 4a, one case with nodal involvement and two cases with distant metastases. As all patients gave informed consent, all patients were still alive at the time of the study. Cases 2, 4 and 5 were initially diagnosed in 2019, case 3 in 2020 and case 1 in 2021. Except the adenocarcinoma of the caecum of case 3 (low-grade, formerly moderate differentiation, G2), all cases were high-grade (formerly poorly differentiated, G3). Cases 4 & 5 were medullary carcinomas of the colon, whereas cases 1 to 3 were adenocarcinomas of different subtypes. The histology (H&E stain) of the MLH1^-^/PMS2^-^/MSH6^-^ cases is shown in **Figure 2** next to IHC staining for MMR proteins of the corresponding cases.

**Table 1:** Clinicopathological features MLH1^-^/PMS2^-^/MSH6- digestive system cancer cases.

Case	Age (yrs.)	Gender	Site	Histology	Differentiation	TNM
**1**	82	F	Gastric antrum	Adenocacinoma, poorly cohesive	High-grade (G3)	pT3, pN0, cM0
**2**	63	F	Ampulla vateri	Adenocarcinoma, mucinous	High-grade (G3)	pT3b, pN0, cM0
**3**	68	F	Caecum	Adenocarcinoma, NOS	Low-grade (G2)	pT2, pN0, cM0
**4**	70	M	Colon transversum	Medullary carcinoma	High-grade (G3)	pT4a, pN1b, cM1(HEP)
**5**	70	F	Left colic flexur	Medullary carcinoma	High-grade (yG3)	ypT4b, ypN0, ypM1(HEP)

F, female; M, male; yrs., years.

None of the patients had another primary tumor or a family history of malignancy at the time the study was conducted.

### Immunohistochemistry and conventional molecular pathology

As already depicted in the study flow chart (**Figure 1**), we could detect five cases with a concurrent loss of immunostaining for MLH1, PMS2 and MSH6 (**Figure 2**). MSH2 staining was retained in all cases. Whereas MLH1 and PMS2 staining was completely lost in all five cases, we could observe only a partial loss of MSH6 expression in cases 1 & 5. In cases 2 to 4, MSH6 staining was lost completely. MSI testing revealed that in cases 1 to 3 both mononucleotide repeats (BAT25, BAT26) as well as all three dinucleotide repeats (D2S123, D5S346 [*APC*], D17S250 [*p53*]) were altered. In case 4, the MSI loci D5S346 (*APC*) and D17S250 (*p53*) were stable, whereas in case 5 only D5S346 (*APC*) was stable. Hence, all cases were considered as MSI-H. Two cases (1&4) showed a hypermethylated *MLH1* promotor indicating a sporadic carcinoma. Additionally, a p.Val600Glu (V600E) mutation could be detected in exon 15 of the *BRAF* gene in case 4. All other cases did not show a *BRAF* V600E mutation. These findings are summarized in **Table 2** besides NGS results of the corresponding cases.

**Table 2:** MMR immunoexpression and molecular characterization of MLH1^-^/PMS2^-^/MSH6^-^ digestive system cancer cases.

Case	MLH1 IHC	PMS2 IHC	MSH2 IHC	MSH6 IHC	MLH1 promotor hypermethylation	MSI-PCR	BRAF status Exon 15	normal tissue germline HRR/MMR variants	tumor tissue somatic HRR/MMR variants
**1**	Complete loss	Complete loss	Retained	Partial loss	Present	MSI-H (5/5)	Wildtype	-	*BRCA2* c.5073delA, p.Lys1691AsnfsTer15 (VAF 30)
									*MSH6* c.3261delC, p.Phe1088SerfsTer2 (VAF 37)
									*MSH6* c.3261dupC, p.Phe1088LeufsTer5 (VAF 26)
									*MLH1* c.1489delC, p.Arg497GlyfsTer11 (VAF 30)
									*TP53* c.216delC, p.Val73TrpfsTer50 (VAF 68)
									*CHEK1* c.728A>G, p.Tyr243Cys (VAF 38)
**2**	Complete loss	Complete loss	Retained	Complete loss	Absent	MSI-H (5/5)	Wildtype	***MLH1*** c.1745T>C; p.Leu582Pro (VAF 52)	***MLH1*** c.1745T>C, p.Leu582Pro (VAF 81)
									*RAD54L* c.374delC, p.Pro125ArgfsTer2 (VAF 34)
									*BARD1* c.1518_1519invTG, p.Val507Met (VAF 99)
									*RAD51D* c.512A>G, p.His171Arg (VAF 33)
									*CDK12* c.544_546delGAG, p.Glu182del (VAF 35)
**3**	Complete loss	Complete loss	Retained	Complete loss	Absent	MSI-H (5/5)	Wildtype	***ATM*** c.4631A>G; p.Tyr1544Cys (VAF 45)	***ATM*** c.4631A>G, p.Tyr1544Cys (VAF 50)
								***FANCA*** c.1771C>T; p.Arg591Ter (VAF 46)	***FANCA*** c.1771C>T, p.Arg591Ter (VAF 52)
									*TP53* c.916C>T, p.Arg306Ter (VAF 27)
									*MSH6* c.3261dupC, p.Phe1088LeufsTer5 (VAF 43)
									*ATM* c.640delT, p.Ser214ProfsTer16 (VAF 25)
									*PALB2* c.1192G>A, p.Val398Met (VAF 32)
**4**	Complete loss	Complete loss	Retained	Complete loss	Present	MSI-H (3/5)	p.Val600Glu	–	*MSH6* c.3227G>A, p.Arg1076His (VAF 18)
									*CHEK2* c.1425del, p.Phe475LeufsTer7 (VAF 18)
**5**	Complete loss	Complete loss	Retained	Partial loss	Absent	MSI-H (4/5)	Wildtype	-	*MSH6* c.3261del, p.Phe1088SerfsTer2 (VAF 27)
									*PALB2* c.839del, p.Asn280ThrfsTer8 (VAF 22)

F, female; HRR, homologous recombination repair; IHC, immunohistochemistry; M, male; MMR, mismatch repair; PCR, polymerase chain reaction; VAF, variant allele frequency in %.

Genes with variants that have not only be identified in tumor but also in normal tissue (**germline**) are highlighted in bold.

Interpretation as well as molecular consequences of the variants are depicted in **Table 3**

### NGS revealing dysregulation of DNA repair in MLH1^-^/PMS2^-^/MSH6^-^ cases

After written informed consent, we performed NGS analysis using AmpliSeq HRR IPMD Illumina custom panel, which is a NGS panel focusing on HRR-related genes. Strikingly, all cases showed relevant variants in HRR genes with a remarkably high variant allele frequency (VAF, median VAF of all reported mutations: 33%) in most sequence variants. NGS analysis results of all five MLH1^-^/PMS2^-^/MSH6^-^ cases are shown in detail in **Table 2**. *A* germline pathogenic variant in MMR genes could only be detected in case 2 (*MLH1* [NM_000249.3]; Exon 16; c.1745T>C; p.Leu582Pro; VAF 52), indicating a hereditary origin of the ampullary cancer in this case. NGS could also reveal germline variant of uncertain significance (VUS) in *ATM* ([NM_000051.3]; Exon 6; c.4631A>G p.Tyr1544Cys) as well as pathogenic variant in *FANCA* ([NM_000135.2]; Exon 19; c.1771C>T; p.Arg591Ter) in our patient suffering from caecal adenocarcinoma (case 3). In both cases, however, no family history conspicuous for a tumor predisposition syndrome could be determined anamnestically. We identified six relevant mutations in cases 1&3, five mutations in case 2 and only two mutations in cases 4&5 in DNA-repair related genes. The affected MMR/HRR related genes were: *ATM, BARD1, BRCA1, CDK12, CHEK1, CHEK2, FANCA, MLH1, MSH6, PALB2, TP53*. Interpretation of the corresponding sequence variants as well as molecular consequences of each variant are depicted in **Table 3**. We further evaluated the genetic content of our observed variants regarding the involvement of mononucleotide repeats (**Table 4**). In case 1 an accumulation of mutations in microsatellites – typical for dMMR cancers – could be observed. To put our findings into a broader context, we extracted the frequency of the detected mutations from publicly available datasets using cBioPortal [https://www.cbioportal.org (36, 37)]. The involved genes that have been mutated in our five cases have already been described for the corresponding malignancies (**Table 5**). Except *TP53*, these genes are mutated in only a small proportion of the respective cancer entities, making their common occurrence even more special (38, 39).

**Table 3:** Detailed overview and significance of MMR/HRR variants observed in MLH1^-^/PMS2^-^/MSH6^-^ digestive system cancer cases.

Case	*Gene*	Variant description HGVS	*Evaluation *ClinVar	*Evaluation *ACMG Classification	Molecular consequence
**1**	*BRCA2* *MSH6* *MSH6* *MLH1* *TP53* *CHEK1*	NM_000059.3:c.5073delA, p.(Lys1691AsnfsTer15)* NM_000179.2:c.3261delC, p.(Phe1088SerfsTer2) NM_000179.2:c.3261dupC, p.(Phe1088LeufsTer5) NM_000249.3:c.1489delC, p.(Arg497GlyfsTer11) NM_000546.5:c.216delC, p.(Val73TrpfsTer50) NM_001330427.1:c.728A>G, p.(Tyr243Cys)	pathogenic pathogenic pathogenic pathogenic pathogenic /	pathogenic pathogenic likely pathogenic pathogenic pathogenic VUS	frameshift frameshift frameshift frameshift frameshift missense
**2**	***MLH1*** *RAD54L* *BARD1* *RAD51D* *CDK12*	NM_000249.3:c.1745T>C, p.(Leu582Pro) NM_003579.3:c.374delC, p.(Pro125ArgfsTer2) NM_000465.3:c.1518_1519invTG, p.(Val507Met) NM_002878:c.512A>G, p.(His171Arg) *NM_016507.2:*c.544_546delGAG, p.(Glu182del)	pathogenic / benign/likely benign / /	/ / / / /	missense frameshift missense missense in frame
**3**	***ATM*** ***FANCA*** *TP53* *MSH6* *ATM* *PALB2*	NM_000051.3:c.4631A>G, p.(Tyr1544Cys) NM_000135.2:c.1771C>T, p.(Arg591Ter) NM_000546.5:c.916C>T, p.(Arg306Ter) NM_000179.2:c.3261dupC, p.(Phe1088LeufsTer5) NM_000051.3:c.640delT, p.(Ser214ProfsTer16) NM_024675.3:c.1192G>A, p.(Val398Met)	VUS pathogenic pathogenic pathogenic likely pathogenic/pathogenic likely benign/VUS [conflicting]	likely benign pathogenic pathogenic likely pathogenic pathogenic likely benign	missense nonsense nonsense frameshift frameshift missense
**4**	*MSH6* *CHEK2*	NM_000179.2:c.3227G>A, p.(Arg1076His) NM_007194.3:c.1425del, p.(Phe475LeufsTer7)	VUS/likely pathogenic [conflicting] /	/ /; **	missense frameshift
**5**	MSH6 PALB2	NM_000179.2:c.3261del, p.(Phe1088SerfsTer2) NM_024675.3:c.839del, p.(Asn280ThrfsTer8)	pathogenic pathogenic	/ /	frameshift frameshift

/= no data available; VUS, variant of uncertain significance.

Genes with variants that have not only be identified in tumor but also in normal tissue (**germline**) are highlighted in **bold.**

Evaluation according to ClinVar and ACMG classification as well as molecular consequence was determined using varsome (https://varsome.com/; accessed on September 25, 2022).

*according to BRCA Exchange also pathogenic (https://brcaexchange.org/variant/623920; accessed on September 25, 2022).

**previously classified as pathogenic according to ACMG criteria in Gieldon et al. (35)

**Table 4:** Genetic content of identified variants regarding mononucleotide repeats in MLH1^-^/PMS2^-^/MSH6^-^ digestive system cancer cases.

Case	Variant	Genetic content of the variant^1^	Mononucleotide repeat in corresponding region of the variant
**1**	*BRCA2* (NM_000059.3); Exon 11; c.5073delA; p.Lys1691AsnfsTer15 *MSH6* (NM_000179.2); Exon 5; c.3261delC; p.Phe1088SerfsTer2 *MSH6* (NM_000179.2); Exon 5; c.3261dupC; p.Phe1088LeufsTer5 *MLH1* (NM_000249.3); Exon 13; c.1489delC; p.Arg497GlyfsTer11 TP53 (NM_000546.5); Exon 4; c.216delC; p.Val73TrpfsTer50 *CHEK1* (NM_001330427.1); Exon 6; c.728A>G; p.Tyr243Cys	TTACTTGAAGC *AAAAAA* ***[A]*** TGGCTTAGAGAAGGAA GCCGGAAGATA *CCCCCCC* ***[C]*** TTCTTAGAGCTTAAAG GCCGGAAGATA *CCCCCCC* ***[C]*** TTCTTAGAGCTTAAAG AAATGACTGCAGCTTGTA *CCCCC* ***[C]*** GGAGAAGGAT TGCCAGAGGCTGCT *CCCCC* ***[C]*** GTGGCCCCTGCACCA AAG *AAAAAAAAA* CAT**[A]**CCTCAACCCTTGGAAAAA	*BRCA2* (NM_000059.3); Exon 11; c.5067_5073 (*A7*) *MSH6* (NM_000179.2); Exon 5; c.3254_3261 (*C8*) *MSH6* (NM_000179.2); Exon 5; c.3254_3261 (*C8*) *MLH1* (NM_000249.3); Exon 13; c.1485_1489 (*C6*) *TP53* (NM_000546.5); Exon 4; c.212_216 (*C6*)
**2**	*MLH1* (NM_000249.3); Exon 16; c.1745T>C; p.Leu582Pro RAD54L (NM_003579.3); Exon 5; c.374delC; p.Pro125ArgfsTer2 *BARD1* (NM_000465.3); Exon 6; c.1518_1519invTG; p.Val507Met *RAD51D* (NM_002878.3); c.512A>G; p.His171Arg *CDK12 (*NM_016507.2); c.544_546delGAG; p.Glu182del	GGTTATCGGAGCCAGCACCGC**[T]**CTTTGACCTTGCCA TTCTGTATGAGCCT *CCCC* ***[C]*** GCTGAGCGCTCATGACC GCAGCCAAGAATGGGCAT**[G]**TGGATATAGTCAAGCT CCGCCTCCTCCAGCTGCTTC**[A]**GGCTAAAACCCAGGA CATCCAAGCTCCACAAG**[GAG]**AAGACCAGGAAAGA	*RAD54L* (NM_003579.3); Exon 5; c.370_374 (*C5*)
**3**	*TP53* (NM_000546.5); Exon 8; c.916C>T; p.Arg306Ter *MSH6* (NM_000179.2); Exon 5; c.3261dupC; p.Phe1088LeufsTer5 *ATM* (NM_000051.3); Exon 6; c.640delT; p.Ser214ProfsTer16 *ATM* (NM_000051.3); Exon 6; c.4631A>G p.Tyr1544Cys *PALB2* (NM_024675.3); Exon 4; c.1192G>A p.Val398Met *FANCA* (NM_000135.2); Exon 19; c.1771C>T; p.Arg591Ter	CCCCAGGGAGCACTAAG**[C]**GAGCACTGCCCAACAAC GCCGGAAGATA *CCCCCCC* ***[C]*** TTCTTAGAGCTTAAAG CAAATTTTTGGAC *TTTTTT* ***[T]*** CCAAGGCTATTCAGT GTATTGGACTTGTTGAAAT**[A]**CTTAGTGATAGATAAC GAAAAACATTCTTGCACA**[G]**TGCCTGAAGGCCTTCT CCCCGCCCTGCTCACACCT**[C]**GAGTGCTCCCCAAAGT	*MSH6* (NM_000179.2); Exon 5; c.3254_3261 (*C8*) *ATM* (NM_000051.3); Exon 6; c.634_640 (*T7*)
**4**	*MSH6* (NM_000179.2); Exon 5; c.3227G>A; p.Arg1076His *CHEK2* (NM_007194.3); Exon 13; c.1425del; p.Phe475LeufsTer7	GGTGATGGTCCTATGTGTC**[G]**CCCAGTAATTCTGTTG TTTCTCTGAGCATAGGAC**[T]**CAAGTGTCACTGAAGGA	
**5**	*MSH6* (NM_000179.2); Exon 5; c.3261del; p.Phe1088SerfsTer2 PALB2 (NM_024675.3); Exon 4; c.839del; p.Asn280ThrfsTer8	GCCGGAAGATA *CCCCCCC* ***[C]*** TTCTTAGAGCTTAAAGG TACTACTCACGACCT *AAAAA* ***[A]*** CATTAGATTTACTTCA	*MSH6* (NM_000179.2); Exon 5; c.3254_3261 (*C8*) *PALB2* (NM_024675.3); Exon 4; c.834_839 (*A6*)

^1^Variants are highlighted in **bold** and [squared brackets], Mononucleotide repeats are indicated underlined and *italic*.

**Table 5:** Frequency of mutations in DNA repair-related genes in different digestive system cancers according to large publicly available datasets.

Entity Dataset	Gene	Percentage (%) of samples with one or more mutations
**Colorectal Cancer** (37)	*TP53*	58.8
TCGA	*MSH6*	4.5
PanCancer Atlas	*ATM*	13.1
534 profiled samples	*FANCA*	4.1
	*PALB2*	2.2
**Gastric Cancer** (37)	*BRCA2*	8.7
TCGA	*MSH6*	3.0
PanCancer Atlas	*MLH1*	2.5
436 profiled samples	*TP53*	48.9
	*CHEK1*	1.4
**Ampullary Cancer** (39)	*MLH1*	0.6
Baylor College of Medicine	*RAD54L*	0.6
Cell Reports	*BARD1*	1.9
160 profiled samples	*RAD51D*	0.6
	*CDK12*	1.3

## Discussion

Immunohistochemistry for MMR proteins (MLH1, PMS2, MSH2, MSH6) is widely used in clinical practice and does not only bear potential implications for the whole family of patients in cases of underlying germline variants but is also of predictive value for response to immune-oncology (IO) therapy (17, 18). Besides usual staining patterns for MMR proteins, there further exist rare staining patterns, which are known to be a peculiar phenomenon, on which there is only limited literature (2–9).

To shed light on the simultaneous loss of expression in both functional MMR heterodimers (MLH1/PMS2, MSH2/MSH6), we retrospectively analyzed 352 cases of different digestive system cancer entities with differing dMMR frequencies (CRC, GC, small bowel carcinoma & ampullary cancer). We could identify five cases with a concurrent loss of MLH1/PMS2 and MSH6 expression. Our study demonstrates that MLH1^-^/PMS2^-^/MSH6^-^ digestive system cancer cases are a rare subgroup of dMMR cancers, that account for 1.4% of all cases in our cohort and do not only occur in CRCs but also in ampullary as well as gastric cancer. As far as we know, this staining pattern has not been described for ampullary or gastric cancer so far. Interestingly, we could only find the concurrent loss of MLH1/PMS2 and MSH6 staining, and no other combination affecting both heterodimers. Even though there are few case studies describing a so-called ‘null-phenotype’ (2, 5), we could not observe a single case with a loss of expression in all four MMR proteins in our cohort. Furthermore, MSH2 expression was retained in all our dMMR cases, which is in line with other studies investigating the exact pattern of MMR expression in dMMR cancer cases (3–5). Besides the rarely occurring immunohistochemical ‘null-phenotype’, the concurrent loss of MLH1/PMS2 and MSH6 expression seems to be the prevalent expression pattern in dMMR cases with an involvement of both MMR subsystems (3–5).

As the concurrent loss of immunoexpression of both MMR heterodimers has not been under much investigation yet, the reasons and the implications of this phenomenon remain mostly unclear. It is already known that neoadjuvant treated tumors can show a scanty MSH6 staining (4, 40, 41), which may explain the partial loss of MSH6 expression in case 5 of our cohort. Moreover, Shia et al. could impressively show that frameshift mutations in the *C8* [NM_000179.2 (MSH6) c.3254_3261] tract of the *MSH6* gene coding region are responsible for scanty MSH6 staining in dMMR cases (4). The same group could not detect this particular *MSH6* exon 5 mutation in neoadjuvant treated pMMR and MSH6^-^ negative CRC cases (4), indicating a different mechanism behind the scanty MSH6 staining in pre-treated pMMR CRCs. As the *MSH6* gene contains a microsatellite of eight mononucleotide repeats (42), it is likely that a secondary somatic mutation in these microsatellites of the *MSH6* gene is a potential mechanism for the complete or partial loss of MSH6 immunoexpression in already MMR-deficient cancers (4). The additional MMR/HRR mutations we could detect in our MLH1^-^/PMS2^-^/MSH6^-^ cases could play a mechanistic role in the loss of MSH6 expression in already MLH1/PMS2-deficient digestive system cancers by paving the way for further mutations in the *MSH6* gene. We also could detect *MSH6* exon 5 mutations in this specific coding region with an involvement of mononucleotide repeats, that have already been described by Shia et al. (4), in three out of our five cases (60%; cases 1, 3 & 5). We additionally evaluated the genetic content of our observed variants regarding mononucleotide repeats as repeats of single bases are the most abundant type of microsatellites in the human genome (43). This was particularly interesting as dMMR cancers are known to be hypermutated (44), and accumulation of mutations in microsatellites is the general main feature of dMMR/MSI-H cancers. Here, the mutations indeed show an involvement of microsatellites - but by far not in all cases (**Table 4**). This phenomenon was particularly pronounced in case 1. Together with the interpretation of the variants and their respective molecular consequence in **Table 3**, this evaluation with regard to the mononucleotide repeats complements the mutation profile of our MLH1^-^/PMS2^-^/MSH6^-^ cases. Together with the *MLH1* promotor hypermethylation, the *MSH6* mutation explains the concurrent MLH1/PMS2 and MSH6 loss in cases 1 and 4. Case 1 presented with an additional sporadic *MLH1* mutation. Besides the *MSH6* mutation, the neoadjuvant treatment in case 5 could be in some part responsible for the partial loss of MSH6 expression in this colon cancer case. In cases 2, 3 and 5, the staining pattern can only be partially explained by our molecular findings. In case 2, we could detect a germline mutation in *MLH1*, that is associated with Lynch Syndrome according to ClinVar. It is already known that ampullary cancers can arise in the setting of Lynch syndrome (45). However, the reported *MLH1* missense mutation only explains for the simultaneous loss of PMS2 but not for the complete loss MSH6 expression. Even though we cannot clarify the loss of MSH6 expression in this case without detected *MSH6* mutations or pre-treatment, it is already known that IHC findings do not always completely overlap with NGS results (46, 47), which may be due to methodical issues like the used NGS panel or the amount of tumor tissue for NGS but also due to biologic reasons, such as epigenetic regulation of expression. The opposite phenomenon would be easier to explain: It is known that some *MLH1* missense mutations lead to catalytically inactive but antigenically active proteins (48, 49). In our subgroup, we could not identify this phenomenon.

To the best of our knowledge, we are the first group to specifically focus on the abnormalities in DNA repair of MLH1^-^/PMS2^-^/MSH6^-^ digestive system cancer cases. Our study provides first evidence that digestive system cancers with this peculiar and rare immunophenotype display widespread dysregulation of DNA repair. Although MMR acts during DNA replication to correct polymerase errors and HRR is responsible for the constant repair of double-strand DNA breaks, these two mechanisms are closely linked to each other as the proteins involved in these processes overlap (25). Cells that have defects in MMR accumulate other mutations such as mutations in HRR-related genes more easily, and vice versa. Therefore, it seems plausible that the loss of expression in multiple MMR proteins may be associated or even rely on more widespread defects in DNA repair. We could detect multiple mutations in HRR-related genes with impressive high allele frequencies in our subgroup. That is why, drugs targeting DNA repair like PARP-inhibitors, that have already found their way into clinical practice in other cancer entities (26, 27), seem to be a logic therapeutic strategy in our MLH1^-^/PMS2^-^/MSH6^-^ digestive system cancer cases as all five cases showed simultaneous mutations (up to 6) in MMR as well as HRR-related genes. We firmly believe working out subgroups likely to benefit from novel therapies, that are currently under investigation and aim to tackle HRR pathway components like *ATM* or *CHEK1/2* (28) – we could detect mutations in all underlying genes –, will be crucial. The successful off-label use of PARP inhibitors in a patient with an *ATM*-deficient CRC has already been described (50). As IHC for MMR is widely available, cost-effective and can give a first hint to a more widespread dysregulation of DNA repair according to our data, our results seem to be of potential clinical significance. The sequence variants we found in genes associated with DNA repair (**Table 2**) are already described in the corresponding cancer entities even though in rare frequency as shown in **Table 5** and further supported by different studies (51–54). Therefore, the potential clustering of such hopefully soon to be treatable mutations in MLH1^-^/PMS2^-^/MSH6^-^ cases is even more noteworthy.

Even though it is already known that dMMR CRCs show a distinct gene mutation profile compared to pMMR CRCs harboring significantly more often *BRCA2* or *ATM* mutations (55), which we also could observe in our MLH1^-^/PMS2^-^/MSH6^-^ cases, we could detect further mutated HRR genes such P*ALB2, RAD51D* or *RAD54L*. These HRR gene mutations, which – as far as we know – have not been linked to dMMR cases previously, seemingly even clustered in our peculiar subgroup. Furthermore, it is known that MSI-H CRCs harbor significantly more mutations than MSS CRCs (44). However, some of our observed mutations have not been linked to dMMR CRCs yet (53, 55) and we could detect up to six synchronous MMR/HRR mutations in our MLH1^-^/PMS2^-^/MSH6^-^ cases, which is something we rarely observe in clinical practice in other cancer entities. Therefore, we believe our findings suggest MLH1^-^/PMS2^-^/MSH6^-^ cases to be a potential special subgroup that that is biologically distinct from dMMR cases with a loss of expression in only one functional heterodimer.

Whereas much is known about the occurrence of germline pathogenic variants in MMR genes in the setting of LS [*see* case 2, *MLH1* (NM_000249.3); Exon 16; c.1745T>C], there is recent evidence that also other cancer susceptibility genes, many of them associated with DNA repair, harbor germline variants in individuals affected by CRCs (51, 52, 56). In case 3, we could observe a pathogenic *FANCA* germline variant as well as a VUS in the *ATM* gene, which are both known to be associated with CRC cases (51, 52, 56). Interestingly, VAFs of the observed germline variants indicate that the MLH1 pathogenic variant (VAF: 81%) is likely a loss of heterozygosity (LOH) event, while the *ATM* VUS (VAF: 50%) and FANCA pathogenic variant (VAF: 52%) do not show evidence of LOH. Yurgelun et al. could show in their large cohort study that more than 6.5% of CRC patients harbor pathogenic germline variants in non-LS cancer susceptibility genes, of which many are involved in DNA-repair processes (52). Even though we only could observe one MLH1^-^/PMS2^-^/MSH6^-^ case with such pathogenic germline variant in a non LS-related gene (case 3, *FANCA)*, it would be highly interesting to correlate these germline variants with immunohistochemical MMR expressions. After all, patients with pathogenic germline variants in DNA-repair related genes are more likely to accumulate additional mutations in MMR proteins, which could eventually lead to a loss of expression in both MMR subsystems. Unfortunately, due to the still relative low occurrence of such germline variants, large numbers of CRC cases, that undergo extensive multigene panel testing as well as immunohistochemical staining for all four MMR proteins, would be extremely beneficial to study this relationship. Such pathogenic germline variants in HRR genes like *BRCA1* should always be considered especially in young CRC cancer patients (57). However, all our patients with MLH1^-^/PMS2^-^/MSH6^-^ cancers were over 60 years of age at time of diagnosis and did not have a familiar history suspicious for cancer predisposing syndromes. Therefore, germline testing would not have been performed outside for research use in these cases.

Our findings seem to be promising considering that dMMR/MSI cancers benefit from IO-therapy and as there is emerging evidence that combining PARP-inhibitors with IO-therapy is reasonable, especially in metastatic disease when there are often few other therapeutic options available: PARP-inhibitors induce double-strand breaks, which promotes neoantigen generation and tumor mutational burden – both enhancing the impact of IO-therapy (58). Furthermore, it has been already shown in a Chinese cohort that CRC patients with HRR mutations show a better survival upon IO-therapy than HRR-wildtype CRC patients (53). To exemplify this idea with regards to our cohort: Case 4 benefits greatly from Pembrolizumab therapy at the moment. In case of tumor progress, the combination of PARP-inhibitors like Olaparib with Pembrolizumab could be a promising therapeutic approach considering the detected *PALB2* mutation in this case because response to PARP inhibition has already been reported for *PALB2*-mutated breast cancer (59). By evaluating pathogenic HRR mutations among different cancer entities in primary as well as metastatic lesions, the already cited study by Heeke et al. could show that most HRR gene mutations displayed either similar (e.g. *CHEK2*, *PALB2*) or even higher frequencies (e.g. *BRCA1/2*) in metastatic than in primary lesions (54). Additionally, responsiveness to PARP-inhibitors has already been shown for CRC cell lines as well as patient-derived organoids (60). This further supports the potential future role of combining IO-therapy with PARP inhibitors in a metastatic setting in MMR/HRR-mutated digestive tract cancers. An interesting still remaining question is whether or not cancers with multiple HRR mutations as our MLH1^-^/PMS2^-^/MSH6^-^ cases are more likely to benefit from PARP-inhibitor therapy than those with only a single HRR mutation. Considering the biologic mechanism of action of PARP-inhibitors, this is indeed conceivable. Apart from that, it should not be neglected that HRR-deficient cancers show an increased sensitivity to conventional DNA-damaging chemotherapy (61, 62), which are often part of the therapeutic strategy in metastatic digestive system cancers depending on the molecular alterations of the tumor as well as patient’s general condition.

The authors are aware of some methodical issues and limitations within this work, namely the absence of copy number variant (CNV) germline analysis for MMR genes and the possibility of mosaicism for some MMR variants. Additionally, non-coding germline or somatic MMR variants that are not covered by the used NGS panel would be also of scientific and potentially clinical interest. Further studies performing CNV analysis as well as whole exome or even whole genome sequencing of MLH1^-^/PMS2^-^/MSH6^-^ digestive system cancers are necessary to fully understand the mutational landscape of these cases.

Of course, our findings are limited by the rarity of MLH1^-^/PMS2^-^/MSH6^-^ digestive system cancers. It would be highly interesting to study more cases in larger cohorts to confirm our findings – especially the accumulation of mutations in HRR genes. Larger case numbers would also be very important to check whether the level of mutations remains stable, as even in our small cohort we found a large range regarding the amount of mutations (two to six simultaneous mutations in HRR genes) – indicating that our subgroup is a heterogenous group as well. Considering the high incidence of cancers arising in the digestive system (19) and the frequency of 1.4% of MLH1^-^/PMS2^-^/MSH6^-^ cases across the included entities, a considerable number of patients could fall into this subgroup overall.

If further studies with larger cohorts can prove the clustering of mutations in DNA-repair related genes in MLH1^-^/PMS2^-^/MSH6^-^ digestive system cancers, this peculiar immunophenotype could immediately trigger additional NGS-testing for HRR mutations as basis for therapy. NGS-based multigene testing is, as far as we know, not routinely performed for all digestive system cancer patients but only occasionally in the specialized setting of molecular tumorboards or in case of suspected hereditary tumor predisposition syndromes. As such extensive multigene testing is still time- as well cost-consuming, a preselection by IHC seems extremely valuable and could be easily implemented into the diagnostic workflow.

To sum up, our study gives first evidence for MLH1^-^/PMS2^-^/MSH6^-^ digestive system cancer cases to be a rare but extremely interesting subgroup that is easy detectable by IHC and may be associated with widespread dysregulation of DNA repair.

## Data availability statement

Further information on the datasets can be obtained from the corresponding author on reasonable request and in restricted form due to privacy reasons. However, all relevant data generated during this study is included in this article.

## Ethics statement

The studies involving human participants were reviewed and approved by the institutional review boards of the Ludwig Maximilian University of Munich (reference: 22-0381). (Chair: Prof. Dr. R. M. Huber Telefon+49 (0)89 440055191 Telefax +49 (0)89 440055192 Ethikkommission@ med.uni-muenchen.de www.ethikkommission.med.uni-muenchen.de, Anschrift: Pettenkoferstr. 8a D-80336 München). The patients/participants provided their written informed consent to participate in this study. Written informed consent was obtained from the individual(s) for the publication of any potentially identifiable images or data included in this article.

## Author contributions

All authors revised the article critically, contributed to it with reflective improvements, and approved the final version. BG, BM, NR, and SD contributed to the study’s conception and design. BG, DV, JW, NR, and SD contributed to the data acquisition process. Finally, BG, BM, NR, and SD contributed to the data analysis and interpretation and wrote the manuscript. All authors contributed to the article and approved the submitted version.

## Acknowledgments

The authors are grateful to Jenny Kleffel, Andrea Seuser, and the whole team of the section molecular pathology of the Institute for Pathology and Molecular Diagnostics, University Hospital Augsburg for their excellent technical assistance.

## Conflict of interest

The authors declare that the research was conducted in the absence of any commercial or financial relationships that could be construed as a potential conflict of interest.

## Publisher’s note

All claims expressed in this article are solely those of the authors and do not necessarily represent those of their affiliated organizations, or those of the publisher, the editors and the reviewers. Any product that may be evaluated in this article, or claim that may be made by its manufacturer, is not guaranteed or endorsed by the publisher.
